# Five Interpersonal Factors Are Predictive of the Response to Treatment of Major Depression With Antidepressants in Primary Care

**DOI:** 10.3389/fpsyt.2018.00416

**Published:** 2018-09-18

**Authors:** José Salazar-Fraile, Ermengol Sempere-Verdú, Santiago Pérez-Hoyos, Rafael Tabarés-Seisdedos, Manuel Gómez-Beneyto

**Affiliations:** ^1^Consorcio Hospital General, Centro de Investigación Biomédica en Red de Salud Mental (CIBERSAM), Valencia, Spain; ^2^Centro de Salud, Paterna, Consellería de Sanitat, Generalitat Valenciana, Valencia, Spain; ^3^Unitat d'Estadística i Bioinformàtica, Vall d'Hebrón Institut de Recerca, Barcelona, Spain; ^4^Department of Medicine, University of Valencia/INCLIVA Health Research Institute and Centro de Investigación Biomédica en Red de Salud Mental (CIBERSAM), Valencia, Spain

**Keywords:** antidepressants, optimism, therapeutic alliance, perfectionism, empathy, social support, primary care

## Abstract

**Introduction:** Factors relating to the interpersonal relationship between the patient and their physician and social environment are important components, which contribute to their response to treatment for major depressive disorder. This study aimed to assess the influence of optimism, perfectionism, therapeutic alliance, empathy, social support, and adherence to medication regimen in the response to antidepressant treatments in the context of normal primary care clinical practice.

**Method:** We conducted a prospective study in which 24 primary care physicians administered sertraline or escitalopram to 89 patients diagnosed with major depressive disorder. The response to treatment and remission of the episode was assessed at 4 and 12 weeks by Cox regression. The effect of adherence to the medication regimen was assessed by multiple regression statistical techniques.

**Results:** Adherence to medication (HR = 0.262, 95% CI = 0.125–0.553, *p* < 0.001) and patient perfectionism (HR = 0.259, 95% CI = 0.017–0.624, *p* < 0.01) negatively predicted the initial response to treatment, whereas patient optimism (HR = 1.221, 95% CI = 1.080–1.380, *p* < 0.05) positively predicted it. Patient optimism (HR = 1.247, 95% CI = 1.1–1.4, *p* < 0.05), empathy perceived by the patient (HR = 1.01, 95% CI = 1001–1002, *p* < 0.05), and therapeutic alliance (HR = 1.02, 95% CI = 1001–1.04, *p* < 0.05) positively predicted episode remission, while patient perfectionism (HR = 0.219, 95% CI = 0.093–0.515, *p* < 0.001) and low adherence to the treatment regimen (HR = 0.293, 95% CI = 0.145–0.595, *p* < 0.001) negatively predicted it. Finally, social support (*p* < 0.01) and therapeutic alliance (*p* < 0.05) predicted adherence to the medication regimen.

**Conclusions:** In addition to taking the antidepressant drug, other factors including the personal interactions between the patient with their primary care physician and with their social environment significantly influenced the patients' initial response and the final rate of episode remission.

## Introduction

Major depressive disorder is a heterogeneous condition characterized by a diagnosis of syndromic validity, without biological specificity. The wide variability of its presentation, course, and responses to different types of treatments is also reflected in the diversity of the theoretical explanations of its etiology, including genetic, biochemical, endocrine and neurophysiological, psychological, and social factors (1). Still other theories deny the validity of the diagnosis from a social perspective (2).

Clinical trials demonstrate the efficacy of drugs in the treatment of depression (1, 3), and studies using rigorous meta-analyses suggest that, based on their efficacy and cost, escitalopram, and sertraline are appropriate choices (4). A recent meta-analisys by the same author added more information for optimal treatment (5). However, non-specific responses to antidepressant pharmacological treatment play an important role in their efficacy. Indeed, a wide range of drugs with demonstrated efficacy also show a high rate of response to placebos (6–9), which has even led some authors to question the use of drugs to treat mild depression (6).

This non-specific effect reflects the serious importance of psychological factors in pharmacological treatments, especially those that entail an interpersonal relationship both inside and outside of the clinical relationship. In the field of clinical care, the concept of placebo has often been used to cover several psychological factors that may be in force in the rendering of antidepressant effects (10). Unlike spontaneous healing, or that produced via pharmacological means, the placebo effect is a form of interpersonal healing (11), which involves both the patient's expectations of their reactions to the drug and the context in which it is administered (12). These factors have led to the study of how various personality traits influence patient responses to placebos, and although the results regarding a wide range of traits such as introversion, intelligence, or self-esteem are controversial, a clear influence has been demonstrated in the case of optimism. In this sense, it has been proven that the relationship between optimism and placebo response is not that of a simple increase or decrease, but rather, that optimism determines the response to the placebo by interacting between the context of the drug administration and the subject's expectations (13, 14). Along with optimism, perfectionism is also important in the response to treatment with antidepressants, especially in terms of how this personality trait interacts with therapeutic alliance. Blat and Zuroff (15) showed that perfectionism significantly influences both psychotherapeutic and pharmacological recovery via antidepressant-mediated treatment by negatively interfering with therapeutic alliance.

In this sense, the therapeutic alliance between the patient and professional at the beginning and throughout the treatment, plays a fundamental role in the outcome of both pharmacological (16) and psychotherapeutic (17, 18) treatments. In the specific case of depression, it is an essential ingredient which is common to the different types of psychotherapy used to treat depression, the placebo effect, and the combination of psychotherapy and pharmacotherapy (19, 20). In pharmacological studies, the effect of therapeutic alliance has always been attributed to its impact on treatment-regimen adherence, specifically to its determining influence on incorrect or insufficient medication use (21). In studies investigating the effect of psychotherapy, in addition to improving adherence, treatment alliance directly and independently influences the outcome of cases with a wide range of diagnoses, populations, and psychotherapy types (17, 22), and this effect increases in relation to the quality of the treatment (18).

The definitions of therapeutic alliance have varied from their initial formulation by Freud (23) to evolve into a concept, which is more universally accepted by various theoretical disciplines. The common factor is an emphasis on collaboration and consensus between the professional and the patient, which is built based on three components: agreement on the therapeutic goals, consensus on the tasks to be performed in the therapy, and the link between the therapist and the patient (24, 25). Several meta-analyses carried out in recent years (17, 18, 22) have coincided in pointing out that therapeutic alliance has a general effect on the outcome of both introspection-based and structured psychotherapies. However, distinction between the three dimensions of therapeutic alliance is important in structured psychotherapy modalities such as cognitive-behavioral therapies. This is because these therapy types emphasize the use of specific techniques and the alliance components, based on their goals and tasks, are more important than in less structured psychotherapies (26).

Because one of the basic components of therapeutic alliance is the emotional bond between the professional and the patient (24, 27), which is favored by mutual understanding (27) or a warm relationship of high regard and respect (28), the ability of the professional to develop and maintain an empathic attitude toward their patient is a fundamental factor in the development of a therapeutic alliance link (29–31) and in its potential influence on the antidepressant pharmacological response. Similarly, among the interpersonal relationship factors that are predictive of a response to antidepressant drugs, social support also stands out in the external field of clinical care (32), given that scarce social support is a risk factor in both the etiology and recurrence of depressive episodes and also in the response to pharmacological treatment (33, 34).

The aim of this study was to investigate the role that the aforementioned psychological factors play in the outcome of antidepressant treatments. Given that antidepressants are mainly prescribed in primary care, we undertook this research in these settings, via family general primary-care physicians (GPs) during the discharge of their daily work and without any external interventions.

## Methods

### Procedure

The study was approved by the ethics committee at Hospital Arnau de Vilanova-Paterna Health Centre and all the patients signed their informed consent; 24 general primary-care physicians (GPs) from the Paterna Health Centre (Spain) participated in the study (mean years of age = 43; mean years working = 21). The Centre has a ratio of 1,680 patients by GP, similar to the rest of the health centers of the Valencian Community and other contexts of the National Health System in Spain. The GPs in the study cover a population of 36,000 adults between 15 and 60 years of age. In the Valencian Community the average of visits to the GP is 4.2 by person year, and 5.7 visits by patient year. In our study, the incidence of depression in primary care associated with the prescription of antidepressants is 4,16 cases by 1,000 patient year, similar, although slightly inferior, to other countries where the figures are between 5 and 10 cases per 1,000 patient year (35).

Patients who were consecutively attended to in the Paterna Health Centre were prospectively selected by their GPs over 12 months during the course of their everyday work. The GPs were asked to select patients with a suspected diagnosis of an episode of major depressive disorder according to their clinical criteria. During the first consultation, the GP provided the patient with information about the disorder but refrained from prescribing treatment and instead arranged a second consultation with them in the following week. At the second consultation, the GPs used the Spanish version of the Patient Health Questionnaire-9 [PHQ-9; (36, 37)], and based on the results, selected patients aged between 18 and 60 years whose PHQ-9 score was equal to or >15. These patients were invited to participate in the study and if they agreed to so do, signed the informed consent form. Those suffering from dementia, bipolar disorder, psychosis, or substance related disorders, as well as those who had less than 12 years' schooling or who were not able to understand the informed consent form or the tests, were excluded. Following the latest update of the literature at the time of the study (5), the GPs established treatment with sertraline (100 mg/day) or escitalopram (20 mg/day) at their discretion and referred the patient for diagnostic confirmation and baseline and follow-up evaluations. The GPs continued to attend to their patients by setting an appointment for the initial follow-up review of the treatment at 4 weeks and undertaking a second follow-up at 12 weeks. Within a period not exceeding 48 h after the GP prescribed the treatment, a psychiatrist confirmed the diagnosis, and a team of two psychiatric nurses trained in the use of the appropriate instruments evaluated the predictive variables. This team was based in a Mental Health Centre adjacent to the Primary Care Centre in Paterna.

### Assessment

The diagnosis was confirmed by using the Spanish version of the Structured Clinical Interview for the Fourth Diagnostic and Statistical Manual of Mental Disorders (DSM-IV) Axis-I Disorders [SCID-I; (38–40)]. Patients who did not meet the SCID-I diagnostic criteria for major depressive disorder were excluded from this study. Those who met the criteria were further assessed, using the procedure and instruments described below.

The PHQ-9 was not only used as a screening tool, it was also employed to decide the outcome variables by evaluating the early response and the clinically-significant improvement in these patients. This questionnaire comprises 9 items derived from the DSM-IV. Its reliability and validity have been proven in different Spanish-speaking countries, both in primary and specialized care (36, 41–43), including when implemented as a telephone interview (42). The threshold for detecting an early response to treatment was established as a reduction of five points between the baseline score and the score at 4 weeks (44–47). At 12 weeks, the clinically-significant improvement was assessed by a 50% decrease the score with respect to the initial evaluation and a score lower than 10 (44–47).

The main difficulty in exploring the patient's evaluation of their GP's attitudes is the strong bias caused by conformism, or social desirability, and impression management. To avoid this problem, GPs' empathy, as perceived by the patients, was assessed using the Repertory Grid Technique [RGT; (48, 49)]. Unlike self-report assessments that only detect explicit attitudes, the RGT also allows assessment of implicit attitudes and is a tool that has been successfully used in a wide variety of contexts (50, 51). For this study a grid of eight elements and five constructs was prepared. The names of the eight most important people in the patient's life were chosen as elements. Following Karkuff's definition of empathy (52), the subsequent sentences referring to the GP's attitudes were used as constructs: “They strive to understand me,” “They understand what I mean,” “They comprehend how I feel,” “They understand me even if I cannot express myself properly,” and “I like my GP”. Empathy scores were obtained by summing the Spearman coefficients of correlation for “I like my GP” and each of the other four constructs.

The therapeutic alliance, as evaluated by the patient, was measured using the Spanish version of the Working Alliance Theory of Change Inventory [WATOCI; (53)]. The WATOCI is based on 17 questions scored on a Likert scale and assesses the degree of agreement between the GP and the patient in terms of the objectives and tasks of the treatment and in the existence of a positive GP–patient understanding (54). The reliability and validity of the Spanish version has been deemed acceptable (55).

Social support was assessed using the Spanish version (56) of the Duke-UNC-11 Social Support Questionnaire (57). This scale evaluates “confidential support” (the availability of people whom the patient can communicate with) and the “emotional support” (demonstrations of love, affection, and empathy) available to the patient. Its test-retest reliability has been verified for both the English and Spanish versions, with scores of 0.9 and 0.8 for the self-administered and professionally-administered versions, respectively. In addition, the validity of the Spanish version has been proven in the contexts of different socioeconomic levels (56, 58). In our study we used a global measure of social support by summing the scores of these scale items.

The optimism trait was measured using the 10-item Revised Life Orientation Test [LOT-R; (59)]. These items, three for optimism, three for pessimism, and four neutral, are measured on a Likert scale. A single score is obtained by summing the optimism item scores and the inverted pessimism scores. We used the Spanish version, which demonstrated similar psychometric characteristics and validity to the original scale (60).

Perfectionism was measured with the perfectionism subscale (61) from the Dysfunctional Attitude Scale [DAS; (62)]. The DAS is a 40-item instrument that expresses cognitive vulnerability to depression and which has shown high reliability and validity (62, 63). Imber (61) revealed that the scale consists of two fundamental factors called perfectionism and need for approval. This distinction has subsequently been validated with other factorial analyses in other samples and has shown good internal consistency and test-retest reliability (61, 62, 64, 65). For this study, the perfectionism subscale was used, considering the total score and the summary in two categories, and defining perfectionists as patients who obtained a score more than one standard deviation higher than the average.

Adherence to the treatment regimen was measured using the Spanish version of the self-reported Morisky Medication Adherence Scale [MMAS-4; (66, 67)]. This scale, which can easily be integrated into clinical consultations, contains four dichotomous items that measure the failure to take medication by evaluating the possible difficulties the patient may refer to, and is scored by summing the items so that the higher the score the greater the non-compliance. Concurrent and predictive validity has shown for the MMAS-4, and it has also been used to evaluate adherence to antidepressant medication in primary care (68, 69).

The SCID-I, PHQ-9, and MMAS-4 scales were all administered at the patient follow-up evaluations.

### Statistical analysis

To analyse the early response and clinically-significant improvement, a Cox regression model was used. As independent variables, the PHQ-9 score was introduced in the first evaluation (to control for the possible confounding effect of the severity of the episode), along with adherence to treatment, empathy, optimism, social support, perfectionism, therapeutic alliance. As the outcome variables, measures of therapeutic change, early response, and clinically-significant improvement were introduced. The outcome variables were analyzed by projecting forward the last observation in the group of patients who agreed to participate in the study and who presented at least one observation after the baseline assessment. To analyse adherence to the medication regimen, we used a multiple regression model in which the same variables indicated above were used as independent variables along with the MMAS-4 score result as a dependent variable.

## Results

**Figure 1:**
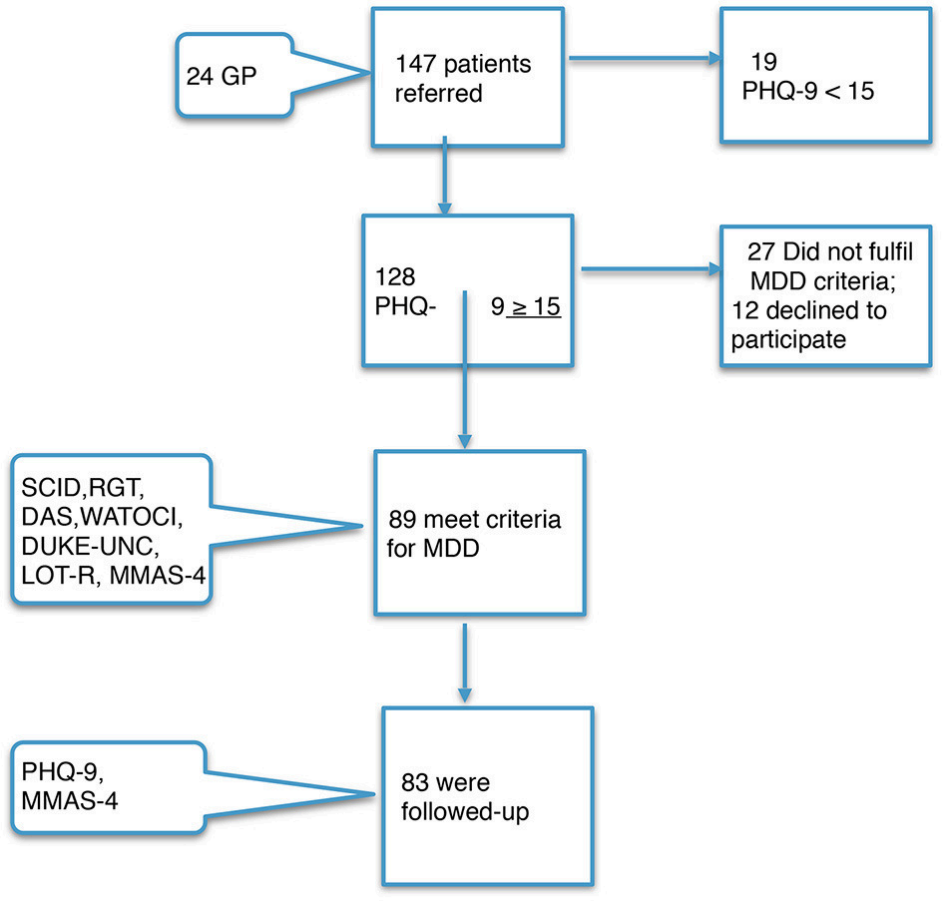
Flow of the patients through the study. GP, General Primary Care Physician; PHQ-9, Patient Health Questionnaire; MMD, Major Depressive Disorder; RGT, Repertory Grid Technique, WATOCI, Working Alliance Theory of Change Inventory; DUKE-UNC, Duke's Social Support Questionnaire, LOT-R, Revised Life Orientation Test; DAS, Disfunctional Attitude Scale; MMAS-4 Morisky Medication Adherence Scale.

The 24 GPs (mean age = 43 years; mean years of working = 21) who participated in the study identified 147 patients with a possible diagnosis of major depressive disorder who were seen consecutively at the Paterna Health Centre over a period of 1 year. Of these, 128 patients presented a PHQ-9 score higher than 15; 39 declined to be included or did not meet the criteria for major depressive disorder or participation in the study, and 89 were finally included; 7% were lost to follow-up (Figure 1). The mean age was 42 years [standard deviation (SD) = 13] and the average level of education was 10 years (SD = 3) of schooling. At the baseline assessment, there were no differences between men and women for any of the variables and there were no significant results in the measurements of response or remission with respect to gender, age, civil status or education-level variables of the patients, nor any of the sociodemographic variables of the GPs (sex, age, or number of years of working). Sertraline was given to 40 (45%) participants and escitalopram was given to 58 (65%) participants. Sertraline did not show statistical differences with respect to escitalopram in the remission or response measurements. No adverse effects due to medication were reported.

Assessment of the early response at 4 weeks (as measured by a decrease in the initial PHQ-9 score of more than five points in the first 6-week interval), indicated that 46 subjects (55.4%) had responded to treatment vs. 37 (44.6%) who had not responded. Of the patients who responded, 7 (15%) did not showed clinically significant improvement. Additionally, 7 (18%) of the patients who did not present an early response, later responded to the treatment. Optimism showed a higher probability of being associated with an early response at 4 weeks (Hazard Ratio [HR] = 1.221, 95% CI = 1.080–1.380, *p* < 0.05), while perfectionism (HR = 0.259, 95% CI = 0.107–0.624, *p* < 0.01) and adherence (HR = 0.262, 95% CI = 0.125–0.553, *p* < 0.001) were negatively associated with the probability of a response. The influence of empathy on social support was not significant, although the effect of therapeutic alliance was near to statistical significance (Table 1).

**Table 1:** Cox regression model of early response to antidepressant treatment.

	**Hazard ratio**	**Sig**.	**95% CI for Exp(B)**	
			**Upper**	**Lower**
Social support	0.972	0.108	0.940	1.006
Optimism	1.221	0.001	1.080	1.380
Therapeutic alliance	1.017	0.074	1.037	0.998
Empathy	1.008	0.207	0.996	1.020
Perfectionism	0.259	0.003	0.107	0.624
Adherence to the medication regimen^*^	0.262	0.000	0.125	0.553

Difference basal phq – first control > 5.

* *Morisky–Green in the first evaluation*.

Regarding clinically-significant improvement at 12 weeks, optimism was associated with a 25% higher probability of remission for each unit increase on the PHQ-9 scale (HR = 1.247, 95% CI = 1.1–1.4, *p* < 0.05). The empathy perceived by the patient (HR = 1.01, 95% CI = 1.001–1.02, *p* < 0.05) and the therapeutic alliance (HR = 1.02, 95% CI = 1.04–1.001) also had a significant effect on the therapeutic outcome at 12 weeks. Perfectionism had a negative influence, meaning that being a perfectionist produced a 4.5-fold increase in the risk that the episode would not subside (HR = 0.219, 95% CI = 0.093–0.515, *p* < 0.001). Similarly, low adherence to the treatment regimen increased the risk that the episode did not improve by 3.4-fold for each unit increase on the PHQ-9 scale (Table 2).

**Table 2:** Cox regression model of clinically-significant improvement considering a decrease ≥50% in the initial score.

	**Hazard ratio**	**Sig**.	**95.0% CI for Exp(B)**	
			**Upper**	**Lower**
Social support	0.980	0.247	0.947	1.014
Optimism	1.247	0.000	1.102	1.411
Therapeutic alliance	1.020	0.039	1.040	1.001
Empathy	1.013	0.035	1.001	1.025
Perfectionism	0.219	0.000	0.093	0.515
Adherence to the medication regimen^*^	0.293	0.001	0.145	0.595

* *Morisky–Green sum of all evaluations*.

Regarding adherence, 72% of the patients did not show any obstacles, or difficulties, to taking their medication, 19.5% had one, and 8.5% had two or more obstacles. There were no differences in the initial severity of the depressive episode, as measured with the PHQ-9 scale, between patients who did or did not abandon their medication regimen (mean abandonment = 20.2, SD = 2.2 vs. mean non-abandonment = 21.7, SD = 2.8; *t* = −1.8; not significant). As shown in Table 3, the multiple regression model analysis showed that therapeutic alliance and social support predicted medication regimen adherence (model *R*^2^ = 0.25, *f* = 3.1, *p* < 0.05). None of the other variables were significant. Gender, marital status, and being on sick leave from work did not have any significant effect on any of the treatment response variables or patient adherence to their medication regimen.

**Table 3:** Linear regression model predicting adherence (non-compliance of medication).

	**B**	***t***	**Sig**.
(Constant)	1.392	2.084	0.043
Social support	−0.025	−2.873	0.006
Optimism	0.038	1.372	0.177
Therapeutic alliance	−0.012	−2.522	0.015
Perfectionism	0.166	0.679	0.501
Empathy	0.002	0.611	0.544

*R^2^ of the model = 0.25, f = 3.1, p < 0.05*.

## Discussion

The most notable result of this present study is that, both the therapeutic alliance and the empathy perceived by patients from their GPs before starting antidepressant treatment for major depression, significantly predict the result of this treatment, regardless of the effect of their compliance to the medication regimen. We also found that the therapeutic outcome is positively predicted by optimism and negatively predicted by perfectionism. This present study explored these factors within the context and conditions of normal primary care clinical practice. Most patients with major depressive disorder are treated within this context (70, 71) but few studies have been performed in this area regarding the influence of optimism, perfectionism, treatment alliance, and empathy, which are all frequently studied in other fields, including in psychotherapy.

It is important to consider that the treatment alliance and empathy perceived by the patient during the study may have been considerably favored by the development of a GP–patient rapport during prior consultations. However, in this study these two factors were assessed only after two consultations had occurred: the depression was evaluated at the first and the medication was prescribed in the second—both before any pharmacological effects could have developed. The temporal precedence between the predictive variables and the results are a crucial condition, thus, given that we met this condition in this study, we can state that our results have high ecological validity and that the direction of the effect is attributable to these variables, independently of the patient's therapeutic regimen adherence.

Our initial hypothesis was that factors related to the personal interaction between the patient and their GP, influence the patient's initial response and their clinically-significant response to treatment, independently of the pharmacological effect. This hypothesis has been corroborated regarding some factors directly related to the GP–patient relationship, although these elements do not all simultaneously exert the same influence. However, outside of this clinical interaction, social support has been shown to influence patient adherence with the medication regimen, specifically in terms of non-compliance.

Both optimism and perfectionism predicted an early response to treatment at 4 weeks and a clinically-significant improvement at 12 weeks. It is important to remember that this was a naturalistic study which evaluated the patients' responses to the prescription of an antidepressant drug by their GP. However, the effects of this intervention are not only rendered by the specific pharmacological effects of the drug administered, but also by a set of actions and interactions between the GP and the patient. This produces a complex mixture of processes in which the choice of drug and its dosage, as well as the GP–patient assessments, agreements, expectations, and attributions all interact in ways which can affect the treatment outcome.

Although our study does not identify any of the mechanisms by which optimism could exert an influence on the therapeutic outcome, optimistic subjects tend to ignore or minimize the contradictions between the expectations of an experience and the experience itself. Thus, in our context, subjects with high levels of optimism who notice an initial change after undergoing an antidepressant drug treatment would likely increase their expectations of improvement, in so facilitating their own response to the prescription (14, 72). Regarding the clinically-significant improvement at 12 weeks, optimistic people tend to more frequently practice healthy behaviors than their non-optimistic counterparts (73), and in addition, optimists tend to have a greater capacity for adaptation and flexibility of thought (74); these abilities mean that these patients would have a greater likelihood of remission from depressive episodes (75).

Perfectionism significantly reduces the outcome of antidepressant and psychotherapeutic treatments, either directly or by conditioning the therapeutic alliance (15, 76, 77). Subjects with high levels of perfectionism tend to find that the degree of change after a therapeutic intervention is insufficient or low; they are more prone to experience dissatisfaction, disappointment, and despair regarding the therapeutic alliance (78) and would cause their therapeutic response to diminish. In contrast, optimism has been shown to reduce the negative consequences of pessimism (79). Optimism guides the focus of attention toward a satisfactory goal; thus, this trait would aid perfectionist subjects in focusing on their improvement, helping them to avoid focusing their attention on censorship or the disappointment of a poorer therapeutic response than they had expected.

Our results indicate that, in addition to the factors that act in the early-response phase, other elements are involved in the clinically-significant response phase. The therapeutic alliance would have a direct influence, and while empathy would also play a significant role as a component of the therapeutic alliance, this trait would be independent from it. It is probable that factors implicated in the therapeutic alliance (24, 25) play a more important role in this second phase. These may include elements related to greater specification of the exact causes of the patient's depression and agreement that certain actions could contribute to the process of resolving the episode. Because the therapeutic alliance is also based on the GP–patient bond, empathy can significantly contribute by generating an environment of support, acceptance, and collaboration, and therefore produce a higher-quality relationship. In addition, empathy also favors the expression of feelings and thoughts (80), thereby helping to clarify problems or conflicts related to the depressive episode.

It is important to consider that in clinical approaches to the administration of a drug, prescription of the drug alone is not usually sufficient for the patient's recovery (81, 82). The process not only involves the prescription, but also the application of procedures to encourage the patient to take the medication and to reassure them about its side effects or inconveniences. These patients also require advice and information about the evolution and prognosis of the disorder. This requires a “minimum therapeutic support condition” (82) like those shared by different psychotherapies for depression—especially the formation of a good therapeutic alliance. Providing a warm therapeutic relationship, a rational explanation about the disease symptoms, and a plan for its treatment help reduce the patient's potential feelings of anguish or demoralization about their condition. Acceptance of pharmacological treatment can be favored by a collaborative environment in which the patient agrees with the doctor that the medication will contribute to improving their depression.

Moreover, an environment of social support can help to resolve and overcome some factors that may lead to the abandonment of medication regimens, such as fear of dependence, difficulty in tolerating the side effects, or lack of the expected response during the first few days. Evidence of the relationship between social support and adherence to treatment regimens has been confirmed by multiple studies and meta-analyses (83). Along with pharmacotherapy and psychotherapy, social support is an important part of treating depression (1). Additionally, in agreement with other studies (84), our results show that social support also influences compliance with antidepressant medication regimens. Guidelines for clinical use indicate that to improve adherence to treatment regimens, clinicians must consider that patients with depression may be unmotivated, pessimistic about their treatment, neglect to properly care for themselves, or suffer from memory problems—all factors that good social support can help to compensate for. Although the processes which influence this effect are unproven, social support acts as a buffer to stress by influencing behavior, favoring adaptive behaviors, facilitating the internalization of norms, and administering sanctions for non-health-oriented behaviors (83). Likewise, the absence of a social support network can interfere with healthy habits, limiting the patient's time and energy, motivation, self-control, confidence, self-esteem, and the management of emotional conflicts.

It is probable that different elements of therapeutic alliance intervene in this second phase. These may include greater specification of the exact causes of the patient's depression, the agreement that certain actions can contribute to resolving the process, as well as a link between the professional and the patient. Our study highlights the significant correlation between the lack of adherence to treatment regimens and the three different factors of the alliance, specifically, with agreement on the goals and causes (*r* = −0.35, *p* < 0.01), tasks and actions (*r* = −0.34, *p* < 0.01), and the GP–patient emotional bond (*r* = −0.29, *p* < 0.05). Other types of support from other areas, such as instrumental social support (e.g., helping to do something or solving social problems), financial support, or the extent and frequency of social network contact, are likely to significantly affect the evolution of the depressive episode. However, these factors are beyond the scope of this study which focused only on the response to treatment with antidepressant drugs administered via primary-care GPs.

This study was carried out in a single health center, limiting the generalization of the final results. Nevertheless, the figures of incidence ratios and the sociodemographic characteristics of the population attended to, suggest that this center is similar to other centers of the Valencian Community and Spain. Further multi-center studies with larger samples of patients and GPs are needed to validate these results. The results cannot be generalized in patients who are suffering from depression and are not identified or treated by GPs. Our results can only be applied to the daily practice of GPs once they have identified and decided to treat a patient. There could also exist an association between the predictive variables and the identification and treatment of the episode. This must be solved in future studies that could clarify whether optimism, perfectionism, alliance, social support and empathy influence the detection and the decision of treating a depressive episode.

We were not able to study whether the predictor variables interact with each other as episodes evolve because these were only recorded at the baseline assessment. Using larger samples in future research would allow different trait subtypes, especially perfectionism, to be distinguished within some variables, and would also allow the influence of different factors, such as alliance, empathy, or social support to be differentiated. This would help to guide specific interventions which could be adjusted to suit the characteristics of each patient. New studies with an experimental design which incorporates a wider variety of drugs would help to further elucidate the probable causal relationship between the variables studied, as well as the role of factors common to the process of prescribing antidepressant drugs.

## Author contributions

JS-F and MG-B contributed to the design, data collection, analysis of results, and writing the final version of the paper. ES-V contributed to the design, data collection and revision. SP-H contributed to the statistical analysis and discussion. RT-S contributed to the design, discussion, and final version of the paper.

### Conflict of interest statement

The authors declare that the research was conducted in the absence of any commercial or financial relationships that could be construed as a potential conflict of interest.
